# Environmental Stability of Crystals: A Greedy Screening

**DOI:** 10.1021/acs.chemmater.1c02644

**Published:** 2022-03-02

**Authors:** Nicholas M. Twyman, Aron Walsh, Tonio Buonassisi

**Affiliations:** † Department of Materials, Imperial College London, London SW7 2AZ, United Kingdom; ‡ Photovaltaic Research Laboratory, Massachusetts Institute of Technology, Cambridge, Massachusetts 02139, United States; § Department of Materials Science and Engineering, Yonsei University, Seoul 03722, Korea; ∥ Singapore-MIT Alliance for Research and Technology, Singapore 138602, Singapore

## Abstract

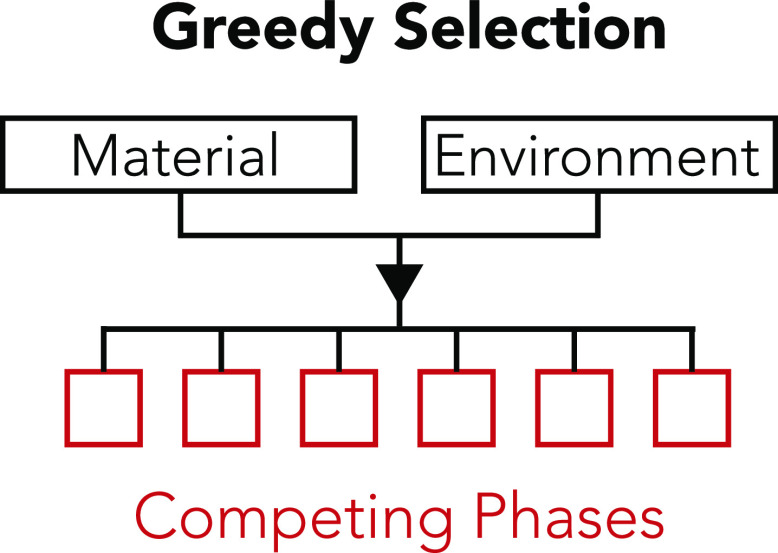

Discovering materials that are environmentally stable and also exhibit the necessary collection of properties required for a particular application is a perennial challenge in materials science. Herein, we present an algorithm to rapidly screen materials for their thermodynamic stability in a given environment, using a greedy approach. The performance was tested against the standard energy above the hull stability metric for inert conditions. Using data of 126 320 crystals, the greedy algorithm was shown to estimate the driving force for decomposition with a mean absolute error of 39.5 meV/atom, giving it sufficient resolution to identify stable materials. To demonstrate the utility outside of a vacuum, the in-oxygen stability of 39 654 materials was tested. The enthalpy of oxidation was found to be largely exothermic. Further analysis showed that 1438 of these materials fall into the range required for self-passivation based on the Pilling–Bedworth ratio.

## I Introduction

Identifying a material for a given application has traditionally been the work of domain experts. However, with the growing size and number of materials databases, researchers are able to find detailed information on the fundamental properties and crystal structures of hundreds of thousands of inorganic materials instantly. Coupled with the sharp increase in popularity of modern data science techniques, this vast resource offers the potential to streamline standard experimental procedures through the introduction of preliminary materials screening.^1−6^

The useful employment of these materials databases largely depends on the individual’s ability to translate material requirements (determined through decades of physical and chemical insights) into searchable, high-fidelity, parametrized “materials descriptors”. Developing good search criteria is a goal shared by many communities, including those who study batteries, thermoelectrics, and solar. If the search criteria are overly stringent, the search will generate many false negatives, excluding viable candidate compounds. Conversely, if the search criteria are too relaxed, the search will generate many false positives, clogging the experimental validation cycle with nonviable candidates. Most proposed materials-search criteria aim to maximize performance using a collection of key parameters: for example, using electrochemical potential to search for new electrode materials and the *ZT* figure of merit to find more advanced thermoelectrics. More rigorously, in the case of solar photovoltaic (PV) materials, we understand that in order to maximize PV device performance a candidate absorber material must exhibit strong optical absorption and long charge-carrier diffusion lengths. These quantities can be parametrized as materials descriptors that include strong optical absorption, large carrier mobility, and evidence of defect tolerance to maximize charge-carrier lifetimes, including large dielectric constant, chemical energy-level alignment, and large bond angles.^7−9^ From these descriptors, lists of candidate compounds with large PV device performance potential can be generated.^8^ Although this approach appears promising conceptually, no 20% efficient device based on such a PV absorber has yet resulted from such a materials search. One possible explanation for this is that no such materials exist. However, this is an unlikely scenario, given the discovery of novel PV absorbers each decade by trial and error and the large number of multinary compounds that some hypothesize are yet to be discovered.^6,10^ A more likely explanation is that our materials-search capabilities have not yet matured to enable true inverse design.

Developing more diverse, selective, and accurate search criteria may assist in narrowing the field of candidate materials. This progression will likely become increasingly useful as modern machine-learning tools are poised to enable more rapid exploration of unexplored multinary spaces, as well as expand existing databases using natural language processing on existing publications.^11−14^ Once improved, these filtering methods will help focus limited experimental bandwidth on the most promising candidate compounds, narrowing the “throughput gap” between theory and experiment.^15^

In practice, making novel materials experimentally is a multidimensional optimization problem, seeking not only to maximize performance but also to optimize factors such as “manufacturability” and “stability”.^16^ The latter of these factors can be broken down into two main subcategories: stability in vacuum and stability in a given environment. Stability in vacuum is typically approximated by estimating the thermodynamic driving force for decomposition in vacuum conditions. This value is found by constructing a convex hull on the energy–composition diagram of the material in question. The convex hull wraps around the energies of all the phases that can be formed from the elements of the initial material, with the ground-state materials lying on this hull. The linear combination of these stable ground-state phases at the original chemical composition may hence be found, indicating how the energy of the system can be minimized. The difference between this value and the material’s own formation energy is the decomposition energy, known as the energy above the convex hull.^17−19^ This value has been used in many studies as a metric to evaluate a material’s vacuum stability, with an energy of 0 meV/atom indicating that the material lies on the convex hull, and is therefore thermodynamically stable with no driving force for decomposition. However, in order to include metastable materials and account for the other factors that influence decomposition, materials exhibiting energy above the convex hull of less than 50–100 meV/atom are typically defined as accessible.^18,20,21^ There are still exceptions to this stability rule in certain chemical systems: most notably nitride systems, which have been shown to have a 90th percentile of metastability at 190 meV/atom.^21^ Despite this, the energy above the convex hull has been used to gauge vacuum stability in many different works and is readily available as a stored materials parameter on the MP database.^17−19,22−24^

This work seeks to expand upon this data-driven approach to stability screening by using a similar approach to estimate the thermodynamic stability of a material in a particular environment.

This environmental stability requirement has been one of the key recurring issues for silicon-alternative solar technologies.^25,26^ For example, in the perovskite solar cell (PSC) field, the success of lead-halide perovskites reaching ever higher power conversion efficiencies is overshadowed by the material’s instability in air, which leads to the production of soluble and toxic decomposition products such as PbI_2_.^26,27^ In mitigation of this issue, manufacturers of PSCs employ expensive device encapsulation, which amounts to a significant portion of the overall panel cost.^28^ An understanding of the driving forces for environmental instability, as well as the potential harmfulness of resultant products, should therefore be considered an essential part of preliminary materials screening.

In some cases, the degradation of the surface of a material can be advantageous for overall environmental stability. This occurs when the formed degradation products act as a barrier layer, preventing further degradation of the bulk. This desirable phenomenon is known as self-passivation and is classically known to be the cause of the air stability of materials like aluminum and silicon. Within the scope of PV materials, the lead-free alternative bismuth-halide perovskites have also been observed to take advantage of this effect, forming a passivating bismuth oxyiodide surface when exposed to air.^29^ Similarly, bare tin monosulfide (SnS) devices have been shown to last two years in air without any efficiency decrease, forming a passivating SnO_2_ layer spontaneously at the device’s surface when exposed to air at ambient conditions.^30−32^ It is difficult to predict whether a material will form these passivating surface layers, due to the variety of factors that influence the nature of the interface formed. Of these factors, the mismatch between the unit cell volumes of the parent and passivating material has been identified to be of particular importance. As first noted by Pilling and Bedworth, this mismatch influences the interfacial stresses at the boundary between the two materials.^33,34^ In their 1923 paper it was postulated that if the molar volume (*V*_*M*_) ratio (shown in eq 1 and known as the Pilling–Bedworth ratio (PBR)) is between 1 and 2, the surface layer will most likely be continuous and therefore passivating.^33^ Outside of this bracket, the interfacial stresses are more likely to lead to fractures and discontinuities in the surface layer, preventing the passivating effect from taking place.^33,34^ This heuristic was first developed for metal oxides forming at the surface of metals but has since been expanded to alloy systems by Xu and Gao.^34^
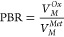
1

Herein, we demonstrate how our stability screening technique can estimate the driving force for degradation of a material in vacuum and in a given environment. The generality of this approach has allowed the algorithm to be tested for accuracy on the conventional energy above the convex hull metric for vacuum stability before being used to create new environmental stability descriptors. We make use of the extensive set of energies in the Materials Project (MP) database.^22,35^ The method also highlights the most thermodynamically favorable degradation products, allowing us to screen materials for self-passivating properties using the PBR. We hope that this work will demonstrate the numerous prescreening possibilities that large open-access databases offer the materials community as well as the power of greedy approaches applied to complex optimization problems.

## II Methods and Results

**Figure 1:**
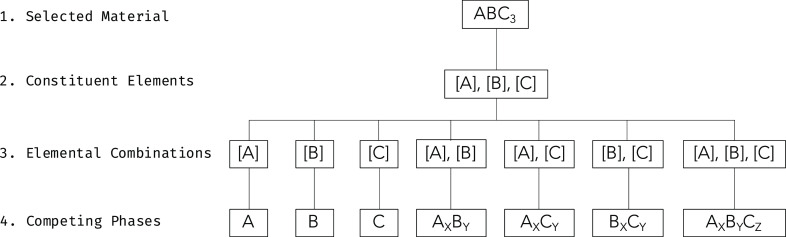
Algorithm flowchart showing the steps that can be taken to find potential degradation products of a material. 1. Initial material selected. 2. Constituent elements of material identified. 3. All possible combinations of these elements found, ignoring order. 4. Database is searched for materials possessing these element combinations.

As previously mentioned, the commonly used stability metric—energy above the convex hull (EATCH)—is equivalent to an energy of decomposition within a closed system. It is calculated by finding the linear combination of phases and compounds that can be formed from the original material composition that minimizes the overall energy of the system. In the context of open-access materials databases, in order to begin evaluating a material’s stability, all possible degradation products must first be compiled. For example, when considering the degradation of the hypothetical material ABC_3_, all materials made up of a subset of these three elements will be evaluated as a possible product of degradation. Figure 1 shows a flowchart for a simple algorithm that can be used to find these potential degradation products using the MP database. The ratio of these materials chosen to calculate the EATCH will depend on their formation energies and compositions with respect to the initial material. In the conventional convex hull method for calculating vacuum stability, the EATCH is found by taking the vertical distance of the original material to the convex hull, wrapping all materials in the composition space on an energy-composition diagram. The corresponding ratio of different stable phases that lead to this EATCH can then be found using a lever rule type approach.

**Figure 2:**
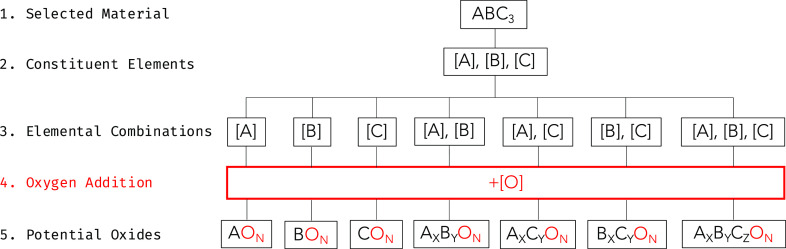
Algorithm flowchart showing how potential degradation products of a material in a particular environment can be found. In this flowchart an oxygen rich environment has been considered. The method is largely the same as that described in Figure 1 with the addition of the environmental elements (e.g., oxygen) to the elemental combinations step.

As with vacuum stability, the first step to evaluating environmental stability is to identify potential degradation products. This can be done on materials databases by a simple extension of the algorithm shown in Figure 1, adding the elements present in the environment to the products search. Figure 2 illustrates this idea, showing a flow diagram to find potential degradation products of *ABC*_3_ in an oxygen environment.

Given the candidates shown in Figure 2, a similar selection process can be carried out based on the formation energies of each material and their compositions. However, to implement this using the convex hull construction adds a significant layer of complexity compared to the original EATCH calculation. This is because this novel scenario requires product materials to be selected independent of the quantity of environmental material within. In vacuum stability tests your selection of degradation products is fully constrained by the constituents of the original material. Therefore, the vertical distance to the hull in the energy composition diagram followed by lever rule can be used to find the driving force for degradation to certain products. Conversely, when evaluating environmental stability, the selection of degradation products is no longer constrained by the constituents of the original material—any amount of the environmental materials may be used. To use a hull method here the hull would therefore need to be constructed on the projection of the energy composition diagram along the axes of the environmental element(s).

For example, when considering the oxygen stability of material AB_2_, the energy–composition diagram of the A_*x*_B_*y*_O_*n*_ system would be used. To find the most stable materials with any amount of oxygen in, a projection must be taken on the O-axis, turning the ternary energy composition diagram into a binary one. The convex hull on this projection (a 1D line in this case) would hence track from A to B, showing the lowest energy materials for any value of *n* (any amount of O). The distance of AB_2_ to this hull would hence give the driving force for degradation, and the corresponding ratio of products could be found by reverting to the initial ternary diagram and performing a lever rule.

Here we demonstrate that the greedy algorithm can be used in place of the extended chemical potential analysis described above to yield an accurate prediction of both vacuum and environmental stability. The underlying approximations are reasonable given the qualitative nature of thermodynamic stability screening, demonstrated by the ∼75 meV/atom EATCH stability tolerance that is the current benchmark for algorithmic stability screening.^18,20,21^

### II.A A Greedy Approach

The task of selecting the thermodynamically optimal potential degradation products and their relative quantities can be considered as a combinatorial optimization problem. The total reaction enthalpy is the value function that must be optimized with respect to the initial material composition (cost function). More specifically, this is a continuous *n*-dimensional knapsack problem, where the number of dimensions, *n*, is defined by the number of elements in the original structure (constraints). The solution to such a problem can be approximated by a greedy algorithm that orders and selects candidate materials based upon a ranking parameter.

**Figure 3:**
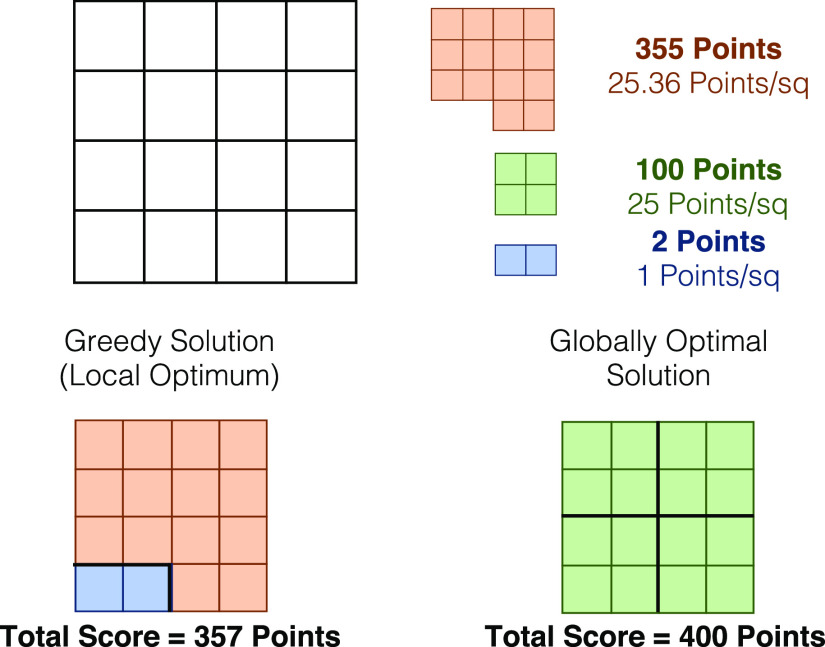
Illustration of grid packing combinatorial optimization problem solved using a greedy approach. The greedy approach will select the red segment first, as this has the highest value density. This forces the next choice to be the blue segment, which has a very low value density. The final solution is therefore 43 points below the globally optimal solution.

Greedy algorithms are commonly used to find approximations to the solutions of complex optimization problems when finding the globally optimal solution is computationally unfeasible. They are based around the principle of continuously taking the locally most optimal “step” in order to reach their final goal. Figure 3 demonstrates how a greedy algorithm can be used to find the locally optimal solution of a combinatorial knapsack optimization problem. In this simple problem, the empty 4 × 4 grid needs to be filled with segments that maximize the overall point score of the filled grid. A greedy approach to solving this problem will sort the segments by a ranking parameter: their value per unit area. This allows segments to be selected in order of their value density. Despite being intuitive and computationally efficient, this approach does not always yield the globally optimal solution, as can be seen in the diagram. To improve upon this shortcoming, greedy algorithms can be run multiple times on the same problem, forcing a different first choice of the algorithm and thus causing a different solution to be returned at each iteration. The best solution from these multiple greedy runs can then be selected to give a more reliable overall solution. Despite this improvement, a local optimum is still found; however, this is often deemed an acceptable compromise in situations where the greedy solution is likely very close to the globally optimal solution.

In the context of stability screening, a similar packing problem is encountered. Once potential degradation products have been identified via algorithms such as those illustrated in Figures 1 and 2, the most energetically favorable combination of these products must be found in order to give an accurate indication of the potential driving force for degradation. The grid being filled in Figure 3 is therefore analogous to a unit of original material that can be “used up” by an array of different degradation products (segments), each with a different formation energy (score). In order to apply a greedy algorithm to this problem, the degradation products need to be sorted by a ranking parameter to allow the most energetically favorable degradation products to be prioritised during selection. This parameter must capture the thermodynamic gain in forming the corresponding material relative to the quantity of original material it uses up, making it equivalent to the score per square metric in the grid example. To construct such a parameter, the stoichiometries of the original and candidate materials were first normalized with respect to the total number of atoms in the unit cell (e.g., *AB* would become *A*_0.5_*B*_0.5_ and *AB*_3_ would become *A*_0.25_*B*_0.75_). The quantity of initial material used by the formation of a unit of degradation material could then be calculated. Dividing the formation energy per atom of the formed material by this amount therefore yields the relative thermodynamic value of forming the product, which can be used as a ranking parameter. This calculation is shown below for the formation of *A*_2_*B*O from *AB* with an energy of −10 eV/atom. Potential degradation products are ordered with respect to this parameter, with the lowest ranking parameter materials being selected first.

1. Normalize Stoichiometries
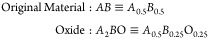
where
2. Calculate Formation Cost, *C*
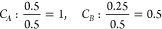
3. Calculate Ranking Parameter
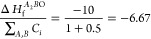

After ranking, the most favorable potential degradation product is then be selected. The maximum amount of this product material that can be formed from the original material is calculated next. Finally, the formation energy for the creation of this quantity of product is logged, along with the remaining amount of original material. This process can then be repeated with the next most promising degradation product, based on its ranking parameter and the remaining material. To improve upon the reliability of this approach, the algorithm is repeated three times for each screened material, forcing the selection of the first, second and third ranked material at the first material selection. The run that yields the most negative reaction enthalpy is then selected as the final result. The enthalpy of reaction per atom in the original material may then be calculated by subtracting the formation energy of the original material from the calculated formation energy of all products.

**Figure 4:**
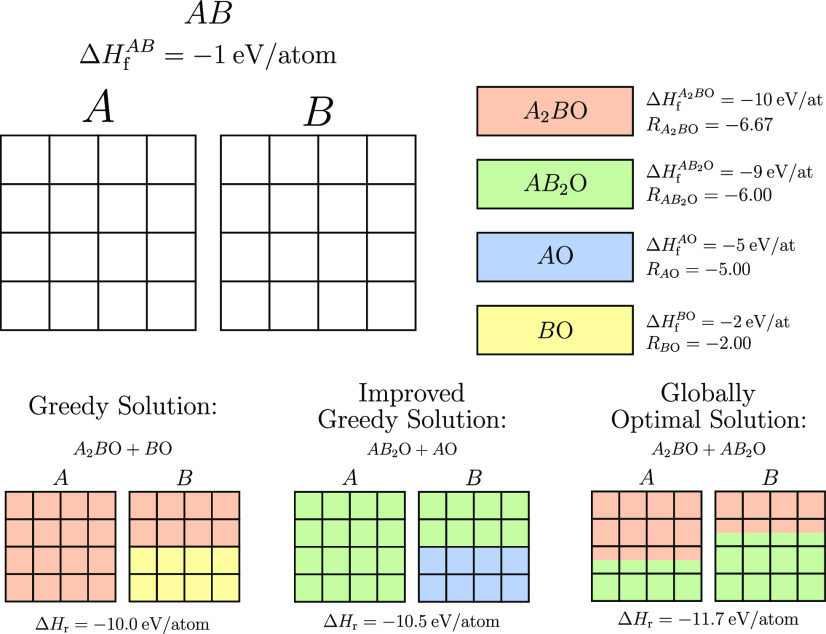
Illustration of a greedy approach to the selection of potential degradation products. The greedy algorithm will first order the possible products by their ranking parameter, which indicates the value–cost trade off of the selection (formation energy per unit of initial material used up). This ordering highlights *A*_2_*B*O as the most favorable first choice, so the maximum amount of this product material is used. This consumes all available units of *A*, leaving half the initial units of *B* available. A degradation product that only consumes units of *B* must therefore be selected next, forcing the unfavorable selection of *B*O. The results of the improved greedy solution are also shown; here the algorithm has been forced to select three different starting products and compare the resulting solutions. Finally, the globally optimal solution is shown, highlighting the potential room for error in this greedy approach. This error is caused by the simplification of this continuous optimization problem to a discretized version.

Figure 4 presents a schematic of this greedy approach to stability screening in a similar format to the previously discussed grid packing problem. In this example, an empty grid has been used to represent the quantity of each element available in the original material. The grids, and hence the material, can then be used up by selecting different amounts of the identified degradation products—in this case oxides. The oxides have been ordered using the ranking parameter, *R*, discussed previously.

As with the example shown in Figure 3, Figure 4 demonstrates the limitations of a greedy approach to this problem by showing how consistently making the locally optimal choice may not yield a globally optimal solution. In this particular scenario, the forced selection process previously described and implemented in the greedy stability screening algorithm would allow the method to find an improved solution of −10.5 eV/atom; however, the globally optimal solution of −11.7 eV/atom would not be found. This is because of the simplification that forces the algorithm to choose the maximum amount of the highest ranked product material, thus discretizing a continuous optimization problem. The globally optimal solution could be found by implementing the hull projection method described previously. It should be noted that the greedy solution will never overestimate the driving force for reaction; therefore, any error can be thought of as a mean underestimation.

## III Decomposition

To evaluate the accuracy of this greedy approach against globally optimal solutions, the algorithm was used to generate estimates for the enthalpy of decomposition of each material in the MP database (see Figure 1)—a proxy for vacuum stability. This value was then compared with the EATCH data of these materials, which is equivalent to the globally optimal solution to their vacuum stability problems. This allowed the mean absolute error (MAE) in the greedy estimate to be calculated.

**Figure 5:**
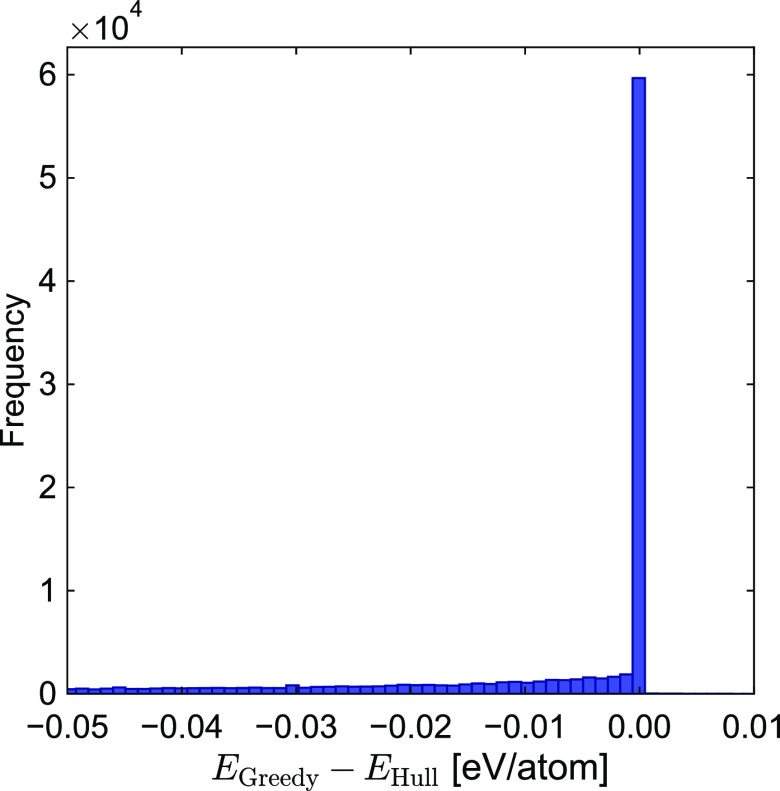
Histogram showing the difference between the enthalpy of vacuum decomposition calculated via the greedy algorithm compared to the energy above the convex hull data of the 126 320 materials in the MP database. *E*_Hull_ > *E*_Greedy_ in all cases.

As previously mentioned, the EATCH metric will have a value of 0 meV/atom when there is no driving force for degradation (a positive enthalpy of degradation in vacuum). All exothermic (and hence thermodynamically favorable degradation reactions) will have an EATCH equal to the absolute value of this enthalpy of degradation in vacuum. To make a comparison between the results of the greedy algorithm and the EATCH values on the MP database, the results of the algorithm were made consistent with the EATCH data. This was done by changing all positive reaction enthalpies to a value of 0 meV/atom to match the “stable” EATCH values and making positive all remaining negative reaction enthalpies. The MAE between the EATCH and greedy data was then calculated and found to be 39.5 meV/atom. This value is well within the MAE between DFT-calculated formation energies and their experimental counterparts, reviewed to be 96 meV/atom by Kirklin et al. with the optimal chemical potential fitting method.^36^ It should be noted that it is unlikely that all of the uncertainty highlighted by Kirklin is attributed to error in the DFT calculations; however, the fact that the MAE of the greedy method is under half of this uncertainty supports the reliability of results yielded from this approach. Moreover, an MAE of 39.5 meV/atom is well within the accepted EATCH stability tolerance of approximately 75 meV/atom, indicating that this method has sufficient resolution to identify thermodynamically stable materials.^18,20,21^ However, in order to improve the reliability of the algorithm when testing for stability in specific environments (Figure 2), only materials within a specified EATCH range were considered for selection. This ensures that only materials deemed stable by EATCH are selected during screening. Figure 5 depicts a histogram of the difference between the values calculated via the greedy and those calculsted via the EATCH methods. As highlighted in the previous section, the greedy estimate cannot be larger than the EATCH value, as the latter value represents the globally optimal driving force, which by definition must be as large as possible. Stability estimates generated using this greedy algorithm will therefore always be conservative, meaning the 39.5 meV/atom MAE can be considered a mean underestimation.

## IV Oxidation Case Study

To demonstrate the application of the greedy algorithm to an environmental stability test, the oxidation of materials in the MP database was investigated. To narrow the testing analysis to materials that are unlikely to spontaneously degrade in inert conditions, only substances with an EATCH value of less than 50 meV/atom were used. This reduced the test set from ∼126 000 to ∼68 000 materials. As the oxidation behavior was being investigated, materials that already contained oxygen were also removed. We thereby exclude cases of partial oxidation. This final adjustment brought the test set down to ∼40 000 materials.

**Figure 6:**
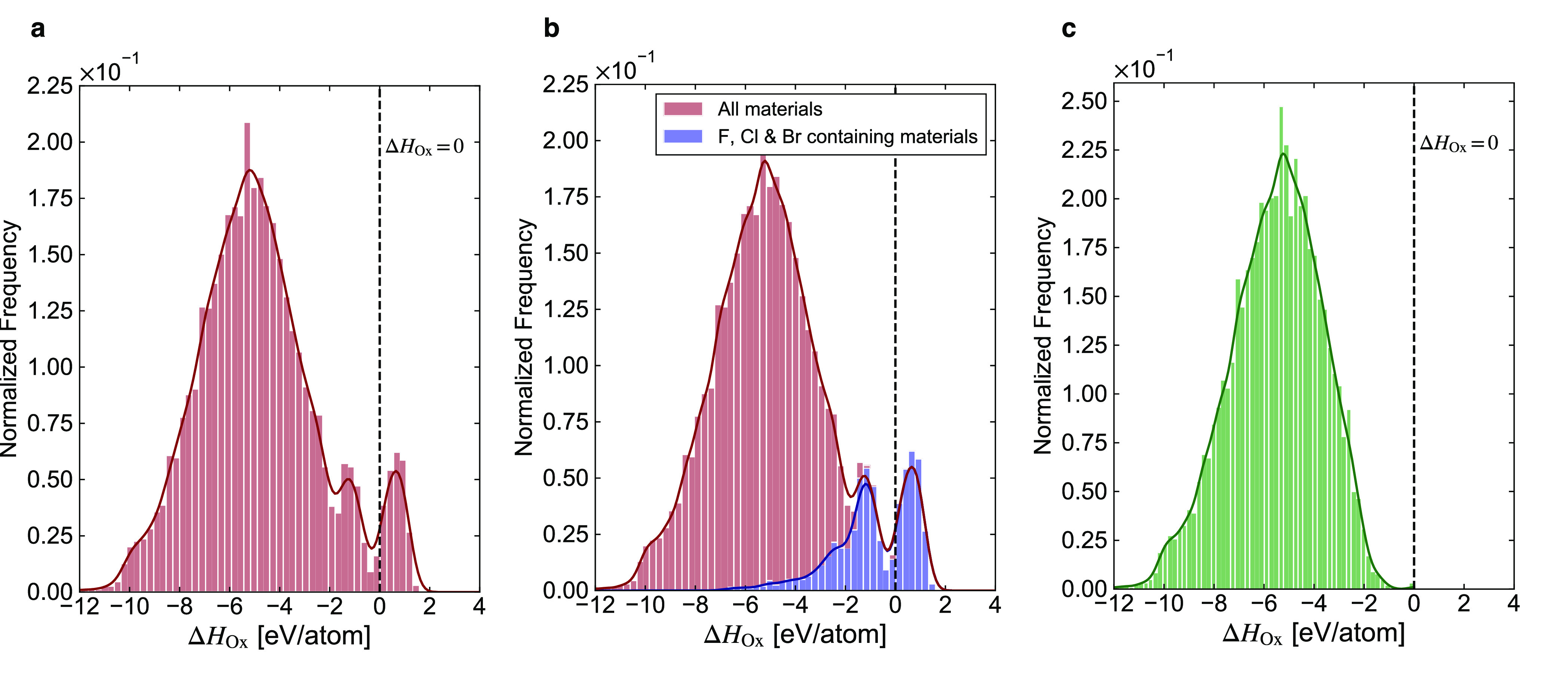
Histograms with kernel density plots of the distribution in the heats of oxidation calculated for the materials on the MP database using the greedy stability tester. a. Distribution of all 39 654 materials tested for oxygen stability. b. Stacked histogram showing the oxidation enthalpies that were yielded from materials containing F, Cl, or Br. c. Histogram of the remaining 33 470 after all F, Cl, or Br containing materials have been removed.

The distribution in the heats of oxidation calculated for these materials using the greedy algorithm is shown in Figure 6a. As expected, most reactions are exothermic, with a symmetric trend to the data below zero. A deviation from this symmetry can be seen by the small peak between −1.4 and −1.2 eV/atom. Investigation into the cause of this unexpected maximum showed that 89% of the materials within the range contained either chlorine or bromine. This is a statistically significant concentration of such materials considering they account for just 8.5% of the whole data set. Inspection of the predicted degradation reactions that led to these oxidation enthalpies showed that in all reactions unusual oxyhalide compounds were formed. These compounds, such as Cl_2_O_7_ and Br_2_O_3_, have an EATCH of below 50 meV/atom so were included in the search for possible degradation products. They also have negative formation energies so were favored by the greedy algorithm once all nonhalide elements were “used-up” in other oxidation reactions. These formation energies are, however, very small relative to the formation energies of other materials on the database. This skewed the data in the histogram and caused the unexpected maximum at a small oxidation enthalpy.

This analysis indicated that other elements that were unlikely to react with oxygen but that displayed stable oxides on the MP database may have impacted the results collected. Fluorine is one such element; with an electronegativity higher than oxygen, it forces oxygen into an unfavorable positive oxidation state. The resulting product, OF_2_, is also shown to be stable using EATCH analysis and has a calculated small negative formation energy. Investigation into these materials showed that all materials that the greedy algorithm had calculated to have a positive oxidation enthalpy were fluorine-containing materials and had yielded a positive oxidation enthalpy due to the small Δ*H*_*Ox*_ of OF_2_. Figure 6b shows a stacked histogram of the enthalpies of oxidation of all F, Cl, and Br containing materials alongside all other materials in the MP database, as calculated by the greedy algorithm. Figure 6c shows that if these materials are removed from the data set, the oxidation enthalpy histogram of the remaining 33 470 materials becomes highly symmetrical with a mean and standard deviation of −5.53 and 1.83 eV/atom, respectively.

The inherently inert noble gases are another category of elements that are highly unlikely to oxidize. The predicted noble gas oxides that are in the MP database therefore exhibit an EATCH outside of the typical stability range causing them to be excluded from the list of potential oxidants during the greedy oxygen stability evaluation. There are, however, a number of other noble gas containing crystals predicted to be stable by EATCH analysis. For these 112 materials, the greedy algorithm failed to run to completion, as the initial material could not be fully used up in reaction with the environmental element (oxygen). This was because any remaining noble gas elements had no stable oxides to degrade into and blocked the total oxidation of the material. Despite this, the calculated Δ*H*_*Ox*_ from these 112 can only be a maximum of 50 meV/atom more positive than the true value (likely much less). This is because the original materials were initially tested for vacuum stability using an EATCH criterion of 50 meV/atom, so any additional degradation of the remnants of original material after the oxygen stability test must have a driving force smaller than this value.

In addition to screening the environmental stability of a material by estimating the driving force for degradation, the greedy stability algorithm returns the materials that are expected to form during this degradation. This data could be of significant value to groups seeking to avoid or encourage the formation of certain materials. To showcase another potential use for this information, the oxidation case-study data was used to find the Pilling–Bedworth ratio (PBR) of the formed oxides. As mentioned in the Introduction, this technique can be used to gauge the likelihood of a material forming a passivating surface oxide—a particularly useful phenomenon for a material to exhibit. It was found that of the ∼33 000 materials tested with results shown in Figure 6c, 1438 degraded to form a set of oxides that all fall into the PBR range, indicating self-passivation. These included materials known for self-passivating in oxygen, such as Al and Zn where low energy Al_2_O_3_ and ZnO phases are identified, respectively. Although PBR has previously been exclusively used to model passivity in metals and alloys, the simplicity of generating this data for screening using this stability algorithm could indicate a potential avenue for further research.

## V Conclusions

In summary, this work presents a method to rapidly screen crystals for environmental stability. The algorithm uses a greedy approach to select the most thermodynamically favorable degradation products that can be formed using substances in the environment. The performance of the algorithm when considering degradation in inert conditions was compared to the energy above the convex hull stability data on the Materials Project database.

Over the 126 320 materials tested, a mean absolute error of 39.5 meV/atom was found between the hull and the greedy data. This resolution is sufficient to identify stable materials given the ∼75 meV/atom tolerance used when categorizing stable materials via methods utilizing DFT-calculated formation energies.^18,20,21^ The greedy estimates of the enthalpy of decomposition, in vacuum or otherwise, will always be smaller than or equal to the true value. This fact makes the MAE equivalent to a “mean underestimation”. To demonstrate an application for the developed screening algorithm, the in-oxygen stability of 39 654 materials was tested. The greedy algorithm successfully assigned heats of oxidation to all materials; however, materials containing F, Cl, or Br were found to significantly skew the otherwise symmetrical distribution of oxidation enthalpies, due to their resistance to oxidation. Once these materials were removed, the distribution in oxidation enthalpies of the remaining 33 470 materials was highly symmetrical about a mean of −5.53 eV/atom. This data was finally used to calculate the Pilling–Bedworth ratios for the materials and the oxides they form, indicating that 1438 were within the range expected for self-passivating materials.

The algorithm proposed here is general and can be used to screen material stability in a variety of environments, which could include an extension to use chemical potentials that account for different gaseous (e.g., partial pressures) and liquid (e.g., pH) environments.

## Notes

The source code for the environmental stability screening algorithm as well as accompanying scripts are available in Zenodo with the identifier https://doi.org/10.5281/zenodo.5110202.
